# Mindfulness for Stress Reduction in Parents of Children with Attention Deficit Hyperactivity Disorder: A Systematic Review

**DOI:** 10.3390/children13070874

**Published:** 2026-06-30

**Authors:** Catarina Lopes, José Tiago Costa-Pereira, Isaura Tavares

**Affiliations:** 1 Department of Biomedicine, Faculty of Medicine, University of Porto, 4200-319 Porto, Portugal; catarina.sousa.lps@gmail.com; 2 Faculty of Nutrition and Food Sciences, University of Porto, 4150-180 Porto, Portugal; jcostapereira@fcna.up.pt; 3 i3S—Institute for Research and Innovation in Health, University of Porto, 4200-135 Porto, Portugal

**Keywords:** mindfulness-based stress reduction, parental stress, attention disorder, mindfulness-based interventions, stress-related outcomes, ADHD

## Abstract

**Highlights:**

**What are the main findings?**

- Mindfulness-based interventions were associated with stress reduction in parents of children with Attention Deficit Hyperactivity Disorder (ADHD) in most studies, with effects frequently sustained or emerging only at follow-up, suggesting that benefits may consolidate over time.
- Beyond stress, the most consistent secondary finding was a reduction in parental over-reactivity, with improvements in psychological well-being and quality of life also observed across some studies.

**What are the implications of the main findings?**

- Mindfulness-based interventions may represent a valid referral option as stress-reduction tools for parents of children with ADHD. However, findings must be interpreted cautiously given the preliminary nature of the existing evidence and risk of bias in the studies.
- Future research should prioritize larger randomized trials, samples with greater involvement of fathers, and longer follow-up periods.

**Abstract:**

**Background/Objectives**: Parents of children with Attention Deficit Hyperactivity Disorder (ADHD) present high levels of stress. This population may benefit from Mindfulness-based interventions (MBI) to reduce their stress. This systematic review assesses current literature about the efficacy of MBI for managing stress and stress-related outcomes among those parents. **Methods**: Studies published up to September 2025 were systematically searched in PubMed, Cochrane Library, Web of Science, and Scopus, and complemented with citation tracking. Both randomized and non-randomized studies were included, provided they quantitatively evaluated parenting stress. Bias assessment was performed using Cochrane ROB-2 and ROBINS-I tools and GRADE analysis was performed. A qualitative synthesis is presented due to the substantial heterogeneity among studies. **Results**: Nineteen studies including nine Randomized Control Trials (RCTs) and ten non-RCTs met the inclusion criteria. Eight studies used an MBI for parents only, ten studies had a parallel intervention for the child/adolescent, and one study used an MBI for parents and teachers. Overall, the studies showed statistically significant stress reduction, either immediately after the end of the MBI (five RCTs and three non-RCTs) and mostly maintained at follow-up or presenting a delayed therapeutic effect which was only evident at the follow-up analysis (one RCT and four non-RCTs). However, some studies reported only mixed findings or no significant differences (three RCTs and two non-RCTs), and one non-RCT reported worsening of stress. Stress-related outcomes varied among different studies. **Conclusions**: MBI may have a significant role in reducing the stress of parents of children with ADHD and may improve stress-related outcomes, such as quality of life, psychological well-being, and parenting over-reactivity. Further studies with longer follow-up periods and lower risk of bias are necessary to clarify possible effects of MBI in stress reduction in parents with ADHD.

## 1. Introduction

Attention Deficit Hyperactivity Disorder (ADHD) is a neurodevelopmental disorder defined by persistent symptoms of inattention, hyperactivity, and impulsive behaviors. The symptoms start in childhood, being mostly identified during elementary school years [1], and occur at levels that impair the normal development/functioning. The disorder is classified into three main subtypes: predominantly inattentive (ADHD-I), predominantly hyperactive-impulsive (ADHD-H), and combined (ADHD-C) [2]. The prevalence of the disorder is estimated to be about 5–11% in children and adolescents worldwide, with a higher prevalence in males, making it one of the most common childhood neurodevelopment disorders [1,2,3].

Besides its increasing prevalence, ADHD is also a current problem due to its considerable burden in the life of children and their families. Children experience poorer academic performance, peer neglect, higher risk of accidental injury and premature death, greater likelihood of conduct disorders during adolescence, and a higher risk of unemployment and antisocial behavior in adulthood, as well as substance use disorders [1]. Due to these constraints and to the frequent burdensome conduct of children [4], their parents face several daily challenges. Worse family function, lower quality of life [5], and experienced greater and significant stress were reported, in comparison to that of parents of typically developing children [6,7]. This is due to multiple causes, namely greater emotional worry for the child due to the disorder. Additionally, the more demanding behavior of the child tends to decrease the time available for the parents and unravels feelings of restricted freedom, being governed by their children’s needs and difficulty in preserving their own identity, as well as higher parental anger [5,6].

Parents also tend to have more difficulty in understanding what their child feels and needs, which can lead to a perception of less emotional closeness with the child and more feelings of sadness, discontent, and incompetence, which in turn can make it harder to perform parental responsibilities [6]. Furthermore, ADHD has a meaningful heritability [1], and parents, apart from helping the child deal with the disorder, may be struggling with their own ADHD symptoms. A financial burden should also be considered due to reduced work efficiency and costs of assistance to the child with ADHD. Families of children with ADHD present with as much as five times more financial burden than in families of typically developing children [8].

Parenting stress may serve as a potential mechanism of mediation for children’s behavioral outcomes and mental health [9], placing parent stress as an important factor to consider in the prognosis of the child health and welfare. Parental stress and related problems, such as psychological well-being and quality of life, should be considered in families of ADHD children, highlighting a need for directed psycho-social support for this specific population.

Mindfulness can be described as the act of purposefully paying attention in the present moment, nonjudgmentally, and the awareness that comes from that practice [10] and can lead to feelings of calm, reduced stress, and self-regulation. Mindfulness-based interventions (MBIs) have demonstrated to be useful in several disorders involving emotional suffering [11,12]. The most widely used MBI is the mindfulness-based stress reduction (MBSR) program, initially proposed by Kabat-Zinn [10,13], and later adapted to the mindfulness-based cognitive therapy (MBCT) program by Teasdale, Segal, and Williams [14]. Whereas neither the MBSR nor the MBCT programs were specifically designed for ADHD, a subsequent program (MYMind program), developed by Susan Bögels at the University of Amsterdam, adapted MBSR and MBCT to ADHD and is distinctive for simultaneous intervention in the child and the parent in parallel sessions [15].

Evidence regarding the use of MBIs for parents is still sparse. To the authors’ knowledge, only two systematic reviews have previously addressed the role of cognitive-behavioral therapies involving MBIs for parents of children with ADHD [16,17]. The results were promising regarding stress reduction but not conclusive. Both systematic reviews included a limited number of studies (six to ten studies) and were limited to studies published up to 2020. Furthermore, these systematic reviews did not provide a comprehensive evaluation of stress-related outcomes, such as well-being, quality of life, mental health and parenting behaviors, which are crucial for the global welfare of families dealing with a child with ADHD. Other limitations of the systematic reviews, pointed out by the authors are the variability in intervention protocols, the high risk of bias, and the limited generalizability and lack of subgroup analyses. Finally, the persistence of effects should be evaluated and most studies used short follow-up periods. Given the growing interest in MBIs in managing parental stress levels and considering that stress-related outcomes were not studied previously, we performed a systematic review aiming to synthesize current available evidence about MBIs in parents of children with ADHD. Specifically, we seek to clarify the role of MBIs for parents of ADHD children and their efficacy in reducing stress and stress-related outcomes.

## 2. Materials and Methods

This systematic review was conducted following the PRISMA (Preferred Reporting Items for Systematic Reviews and Meta-Analyses) 2020 Statement [18], and the research protocol was registered on PROSPERO (ID: CRD420251231971).

The review addressed the following PICO research question: In parents of children/adolescents with ADHD (P), do mindfulness-based intervention (I), compared with any comparison condition (e.g., waitlist control, usual care, active control, baseline measurement) (C), reduce parental stress and improve stress-related outcomes, such as well-being, quality of life, mental health, self-compassion, parenting behavior, and parental ADHD symptoms (O)?

### 2.1. Eligibility Criteria

Studies were considered eligible for inclusion if a Mindfulness-Based Intervention was used on parents of children and adolescents with ADHD or on both parents and child/adolescent. Including quantitative measures of parental stress or quantitative measures of parental stress and stress-related outcomes. Both randomized and non-randomized published studies were included, if in the English language. They were compared with a control group, a baseline measure, or a waitlist control.

The exclusion criteria included studies in which the intervention was not mindfulness-based, that did not include parents of children with ADHD (e.g., only focused on the child, general parent population, parents of children with other neurodevelopmental disorders that were not ADHD), and studies that did not report parenting stress or stress-related outcomes quantitatively. Other reasons for exclusion were a non-trial study design (e.g., study protocols, reviews, book chapters, case reports) or the study being written in a language other than English.

### 2.2. Search Strategy

A systematic literature search was conducted in four electronic databases: PubMed, Cochrane Library, Web of Science, and Scopus. The search comprised articles published from inception to the date of the last search, 10 September 2025, across all four databases. The search was run with the search terms: Mindfulness AND (ADHD OR Attention Deficit Hyperactivity Disorder) AND (parent OR parents OR parental) AND stress. A full search strategy is available in Appendix A.

Additional literature was identified by citation tracking of included studies’ reference lists and considered in the study if eligible.

### 2.3. Data Management and Selection Process

All references were imported and managed with the aid of the Rayyan electronic platform. After duplicate studies were removed, the studies were screened by title and abstract for eligibility, and eligible articles were analyzed in full text for final inclusion. The selection process of the studies was performed by two independent reviewers.

No automation tools were used for the decision. In case of a lack of agreement in the selection, both reviewers discussed among themselves, and if necessary, a third reviewer arbitrated until a consensus was reached.

**Figure 1:**
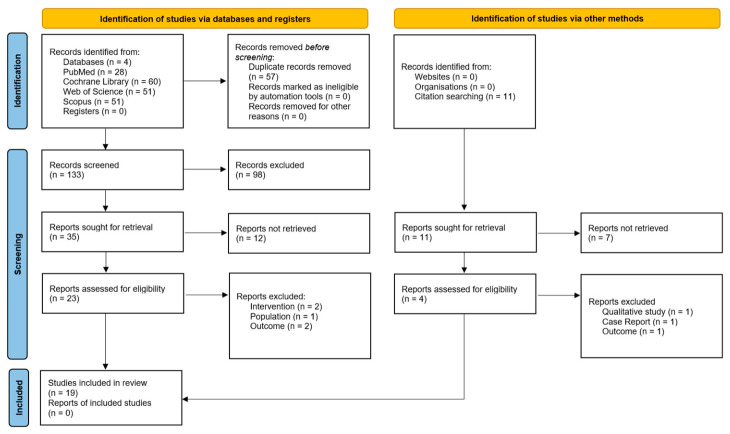
PRISMA 2020-compliant flow diagram of the study selection process, including identification, screening, eligibility, and inclusion of studies.

The study selection process and results are registered in the PRISMA 2020 flow diagram for new systematic reviews, including searches of databases, registers and other sources (Figure 1).

### 2.4. Data Collection

Data regarding the study was collected as described in the Appendix B, and organized in a spreadsheet table, according to the following: Study details (study reference, year of publication, study type, location); ADHD children (mean age, age range, medication status, ADHD subtype); Parents (mean age, gender, marital status, employment and education); MBI (type of intervention, duration); Control (type of control, duration); Outcome (stress measure, stress-related outcomes measure, points of time of assessment, results).

All data was extracted by one independent reviewer and checked by another independent reviewer. No additional information was obtained or confirmed from the studies’ investigators; if the data in the study’s publication was unavailable or unclear, it was not included.

### 2.5. Risk of Bias Assessment and Certainty Evidence

Following data extraction, the risk of bias in the included studies was assessed using the ROBINS-I tool for non-randomized studies of interventions [19] and the Cochrane RoB2 tool for randomized trials [20]. Briefly, the ROBINS-I tool evaluates seven domains of bias: confounding, selection of participants into the study, classification of interventions, deviations from intended interventions, missing data, measurement of outcomes, and selection of reported results. Each domain is rated as low risk (green), moderate risk (yellow), serious risk (red), or critical risk (maroon), with signaling questions guiding judgments to inform an overall risk of bias rating.

The RoB2 tool addresses five domains: bias arising from the randomization process, deviations from intended interventions, missing outcome data, measurement of outcomes, and selection of reported results. Domains are judged as low risk of bias (green), some concerns (yellow), or high risk of bias (red).

Risk of bias plot displaying domain-level and overall judgments was generated using the Risk of bias VISualization (robvis) [21].

The certainty of the evidence was assessed using the Grading of Recommendations, Assessment, Development, and Evaluations (GRADE). The GRADE includes five domains: Risk of bias, Inconsistency, Indirectness, Imprecision, and Publication bias. The overall certainty of the evidence was scored as high, moderate, low, and very low [22].

### 2.6. Data Synthesis

Study characteristics were systematically summarized using summary tables, which integrate key extracted parameters such as study location and design, parental demographics (age, gender, marital status, employment, and education), child demographics (age, gender, medication status, ADHD subtype), intervention details (type of MBI and type of control), methods of stress measurement, and other relevant variables obtained during data extraction.

Due to substantial heterogeneity among the included studies in terms of study design, participant characteristics, interventions, and effect estimates, a meta-analysis was not performed. Instead, a structured qualitative synthesis is presented for stress measurement results and stress-related outcomes. Statistical significance was considered at *p* ≤ 0.05.

Effect sizes (ES) were reported using different metrics across studies, including Cohen’s d and its variants, e.g., dz (standard interpretation as 0.2 small, 0.5 moderate, and 0.8 large), and eta squared (η^2^) or partial eta squared (ηp^2^) (standard interpretation as 0.01 small, 0.06 moderate, and 0.14 large).

## 3. Results

### 3.1. Study Selection

The results of the search and selection process for eligible studies are described in the PRISMA 2020 flow diagram for new systematic reviews, which included searches of databases, registers and other sources [23] in Figure 1.

A total of 190 studies were identified via database search, of which 91 were identified as possible duplicates in the Rayyan electronic platform and were reviewed individually by one independent reviewer, resulting in a total of 57 duplicate articles removed.

The selection process for the remaining 133 studies was performed by two independent reviewers based on the title and abstract. Of these, 110 studies were considered non-eligible for inclusion, and a total of 23 articles proceeded to full-text screening.

At full-text screening, five studies did not meet the selection criteria due to not performing a mindfulness intervention, a lack of presentation of results for parents, and a lack of direct assessment of stress. The remaining 18 were considered eligible for inclusion, and via citation tracking of these studies, an additional study [24] was identified and included after full-text examination.

A total of 19 studies [24,25,26,27,28,29,30,31,32,33,34,35,36,37,38,39,40,41,42] were included in this systematic review.

### 3.2. Study Characteristics

This review includes nine Randomized Control Trials (RCTs), three Quasi-experimental Studies, and seven pre–post intervention studies, as described in Table 1.

Most participants were mothers with ADHD sons; refer to Table 2 for more demographic details.

Most studies used weekly group sessions of MBIs, such as the MYmind protocol. Interventions were predominantly delivered in face-to-face group formats, with sessions ranging from one to three hours in duration, with fewer interventions delivered online [30,32] or as self-directed interventions [25].

Most MBI programs had an 8-week duration, but interventions lasting for 4 weeks [32], 5 weeks [30], 6 weeks [33] and 12 weeks [34] were also identified. One study did not report the duration of the intervention [24].

Eight studies used an MBI for parents only, while ten studies also had a parallel intervention for the children or adolescents (Table 3), and one study used an MBI for parents and teachers [35]. As for control, three studies used a usual care-only control group, six used a waitlist control group, two used active control, and eight used baseline control.

The characteristics of each included study population are listed in Table 2, and the respective intervention/control and assessment timepoints are described in Table 3. Information regarding parents’ age, gender, marital status, education and employment, children’s age, gender, medication and ADHD subtype, study intervention and control, and assessment time was detailed when information was available in the studies.

### 3.3. Stress Outcome

Parental stress was assessed across studies with several validated measures. The most common was the Parenting Stress Index (PSI), used in 14 of the studies, followed by the Perceived Stress Scale (PSS) used in 3 studies. Apart from them, the Parental Stress Scale (ParSS), Depression Anxiety Stress Scale (DASS-21), and Stress Index for Parents of Adolescents (SIPA) were also used in individual studies. The stress measurement tools and timepoints are presented in Table 3.

Follow-up assessments ranged from a month to a year after MBI. We considered short-term follow-ups as less than 8 weeks post-intervention, mid-term for follow-ups equal to or greater than 8 weeks but less than 6 months, and long-term for follow-ups of 6 months or more after MBI (Table 3). Several studies used a mid-term follow-up assessment [26,27,30,32,37,39,40,41], and some had a long-term follow-up [27,37,38,41]. Additionally, two studies assessed short-term follow-ups [28,29].

**Table 3:** Characteristics of the interventions and stress measurements (tools and timepoints). Studies are ordered in accordance with Table 1.

Reference	Intervention		Control	Stress Measure	Assessment Timepoints
	Program	Sessions			
Behbahani et al. (2018) [26]	Mindful Parenting Training for parents	8 weeks, 1.5 h weekly sessions	CAU	PSI	Baseline Post-intervention Mid-term follow-up (8-week)
Lo et al. (2020) [33]	Family MBI for parents and children	6 weeks, 1.5 h weekly sessions	Waitlist	PSI	Baseline Post-intervention
Liu et al. (2021) [31]	Mindful Parenting Program for parents	8 weeks, 3 h weekly sessions	Waitlist	PSI	Baseline Post-intervention
Mah et al. (2021) [34]	MBPT for parents	12 weeks, 2-hweekly sessions	SBPT	PSI ^1^	Baseline Post-intervention
Siebelink et al. (2022) [37]	MBI “MYmind” + usual care for parents and children	8 weeks, 1.5 h weekly sessions + booster session 8 weeks later	CAU	DASS-21	Baseline Post-intervention Mid-term follow-up (2-month) ^2^ Long-term follow-up (6-month)
Valero et al. (2022) [38]	MBI “MYmind” for parents and children	8 weeks, 1.5 h weekly sessions	Waitlist	PSI	Baseline Post-intervention Long-term follow-up (6-month)
Lo et al. (2024) [32]	Online mindfulness-based program for parents	4 weeks, 1 h weekly online session + 15–20 min short video sessions each weekday	Waitlist	PSI	Baseline Post-intervention Mid-term follow-up (2-month)
Wong et al. (2024) [41]	MBI “MYmind” for parents and children	8 weeks, 1.5 h weekly sessions	CBT	PSI	Baseline Post-intervention Mid-term follow-up (3-month) Long-term follow-up (6-month)
Law et al. (2025) [29]	MBSR for parents	8 weeks, 2.5 h weekly sessions + retreat	CAU	PSS ParSS	Baseline Post-intervention Short-term follow-up (1-month)
Van der Oord et al. (2012) [40]	Mindful Training for parents and children	8 weeks, 1.5 h weekly sessions	Waitlist	PSI	Baseline Post-intervention Mid-term follow-up (8-week)
Bakhshayesh et al. (2015) [24]	Mindfulness Training for parents and children	8 sessions, 1.5 h weekly sessions	Baseline and between intervention groups	PSI	Baseline Post-intervention Follow-up
Bögels et al. (2021) [27]	MBI “MYmind” for parents and children	8 weeks, 1.5 h weekly sessions + follow-up session 8 weeks later	Waitlist	PSI	Baseline Post-intervention Mid-term follow-up (8-week) Long-term follow-up (1-year)
Van de Weijer-Bergsma et al. (2012) [39]	Mindful Training for parents and adolescents	8 weeks, 1.5 h weekly sessions + booster session 8 weeks later	Baseline	PSI	Baseline Post-intervention Mid-term follow-up (8-week) Mid-term follow-up (16 weeks) ^3^
Haydicky et al. (2015) [28]	MBI “MYmind” for parents and adolescents	8 weeks, 1.5 h weekly sessions + booster session 6 weeks later	Baseline	SIPA	Baseline (4 weeks before) Pre-intervention (Day 1) Post-intervention Short-term follow-up (6-week)
Anderson and Guthery (2015) [25]	Mindfulness-Based Psychoeducation for parents	8 weeks, reading at own pace “Everyday Blessings: The Inner Work of Mindful Parenting” by Myla and Jon Kabat-Zinn	Baseline	PSI	Baseline Post-intervention
Miller and Brooker (2017) [35]	Modified MBSR for parents and teachers	8 weeks, ≤2 h weekly sessions + retreat	Baseline	PSS	Baseline Post-intervention
Zhang et al. (2017) [42]	MBI “MYmind” for parents and children	8 weeks, 1.5 h weekly sessions	Baseline	PSI	Baseline Post-intervention
Leitch et al. (2023) [30]	Online MBI-Parents that Mind App for parents	5 weeks, 1–2 h weekly home practice via App + 2 Face-to-Face retreats	Baseline	PSI	Baseline Post-intervention Mid-term follow-up (8-week)
Rice et al. (2023) [36]	MBSR for parents	8 weeks, 2 h weekly sessions + retreat	Baseline	PSS	Baseline Post-intervention

CAU: Care As Usual; MBPT: Mindfulness enhanced Behavioral Parent Training; SBPT: Standard Behavioral Parent Training; CBT: Cognitive Behavioral Therapy. PSS: Perceived stress scale; ParSS: Parental stress scale; PSI: Parenting Stress Index; DASS-21: Depression Anxiety Stress Scale; SIPA: Stress Index for Parents of Adolescents. ^1^ Only two out of the three subscales were used. ^2^ DASS-21 was not assessed in the 2-month follow-up. ^3^ Due to large drop-out, data at 16-week follow-up was not analyzed.

Most studies reported a significant reduction in parental stress at the post-MBI timepoint (Table 4). Significant improvements were observed in several RCTs, namely references [26] (*p* < 0.001), [33] (*p* = 0.001, d = 0.19), [31] (*p* = 0.004, d = 0.44), [38] (*p* = 0.038, ηp^2^ = 0.155), and [29] (*p* < 0.001, η^2^ = 0.192 for PSS and η^2^ = 0.599 for ParSS). Several non-RCTs also found significant stress reductions at post-intervention, namely references [24] (*p* ≤ 0.001), [25] (*p* = 0.018), and [36] (*p* = 0.03).

Some studies reported mixed findings. For instance, a significant stress reduction was detected in fathers (*p* < 0.01, d = 0.7) and not in mothers [39]. A study [28] did not report a significant total stress score reduction at post-intervention, but showed significant stress reduction in parameters, such as stress related to social isolation/withdrawal and life restrictions. A significant stress reduction was reported in both the intervention (*p* < 0.001) and control groups (*p* < 0.003), but greater among parents in the intervention group, with significant time-by-group interactions (*p* < 0.001) [26].

Improvements in stress were maintained at follow-up, namely in several studies namely references [26] (*p* < 0.001), [29] (*p* < 0.05, η^2^ = 0.192 for PSS and η^2^ = 0.599 for ParSS), [24] (*p* ≤ 0.001), and [39] (*p* < 0.01, d = 1.1). The persistence of effects was not found in the follow up a study [38], (*p* = 0.067, ηp^2^ = 0.128). For the remaining studies, the follow up was not assessed [25,31,36].

Some studies did not show significant differences at post-intervention but achieved significant stress reduction at follow-up assessments. It was the case of one of the RCTs [37], which had significant stress reduction at long-term follow-up (6 months), in both Intention-to-treat (ITT) and Per Protocol approaches (*p* < 0.05, d = 0.42 and d = 0.51, respectively). Additionally, from the non-RCT study [40] significance was reached at mid-term follow-up (*p* < 0.01, ES = 0.57). Other study [27] did not reach significance at post-intervention nor at mid-term follow-up, but reached significant improvements of small effect size at long-term follow-up (one year) (*p* ≤ 0.001). Two studies reached significant improvements of total stress reduction at short-term follow-up [28] (*p* = 0.010, d = 0.81), and at mid-term follow-up [30] (dZ = 0.98).

Nevertheless, some studies did not report statistically significant change in stress outcomes, namely RCTs [32,34,41] and non-RCT [35] studies. The lack of significance persisted at mid- to long-term follow-up. Two studies did not have a follow-up assessment [34,35]. Additionally, one non-RCT [42] showed significant worsening in stress from baseline to post-intervention (*p* = 0.01, ES = −0.18).

### 3.4. Stress-Related Outcomes

Some studies also examined parameters other than stress scales. These parameters account for stress-related outcomes, and the results are summarized in Table 4.

Regarding quality of life (QOL) and well-being, a general trend of significant post-intervention improvements on the 5-item World Health Organization Well-Being Index (WHO-5) was observed the studies of Lo et al. (2020) [33] (*p* = 0.005), Wong et al. (2024) [41] (significant within-group improvement, *p* < 0.001, but no significant between-group difference compared with the active control group), and Siebelink et al. (2022) [37] (*p* < 0.01 in ITT and *p* < 0.05 in Per-Protocol). The maintenance of these improvements varied and a study showed significant gains at both 3- and 6-month follow-up (*p* = 0.006 and *p* = 0.039, respectively) [41] and another study [37] did not demonstrate significant effects at 6-month follow-up.

Two other studies [29,36] sought enhanced quality of life following MBI, using the World Health Organization Quality of Life-Brief (WHO-QOL-BREF). The study of Law et al. (2025) [29] found significant improvement in the psychological domain (*p* < 0.001), social relationships domain (*p* < 0.05), and environment domain (*p* < 0.001), and no significant differences in overall quality of life, overall health, or physical health. Rice et al. (2023) [36] showed significant improvements in the social relationships domain (*p* = 0.021) and the overall total score (*p* = 0.016), but differences were not significant for the other domains of the scale (physical, psychological, and environmental).

Additionally, mediation analyses in Lo et al. (2020) [33] indicated that improvements in child ADHD symptoms significantly mediated the improvements in parent well-being.

In terms of psychological effects, significant improvement in depressive symptoms were reported (*p* = 0.001) in Liu et al. (2021) [31]. Leitch et al. (2023) [30] reported a significant reduction in psychological distress post-intervention (dZ = 0.72) and at follow-up (dZ = 0.72). Variable effects in anxiety symptoms were reported. A decrease in anxiety symptoms after intervention was detected [35] (*p* = 0.04, d = 0.53), whereas the other study [31] reported significant decreases in both the intervention (*p* = 0.003) and control (*p* = 0.003) groups after the intervention. Lo et al. (2024) [32], despite showing positive changes in depression and anxiety, had similar changes in the waitlist group and no significant time-by-group interaction effects. Some studies did not report significant effects in mental health, namely depressive symptoms [35,37].

Improvements in self-compassion in the intervention group were reported by Liu et al. (2021) [31] (*p* < 0.001, d = 0.62) at post-intervention. Additionally, the study of Siebelink et al. (2022) [37] did not show significant differences in the results at post-intervention but showed significant improvement at the 6-month follow-up, both for ITT (*p* < 0.01) and per-protocol (*p* < 0.001) approaches. A correlation analysis showed that improvements in self-compassion were associated with improvements in children’s ADHD scores [31].

Dysfunctional parenting was assessed by the Parenting Scale (PS) in several studies [27,30,34,38,39,40,42], using certain or all subscales of the PS (laxness, over-reactivity, and verbosity) with results differing among studies. A significant main effect in the intervention group (*p* < 0.001, η^2^ = 0.312) and a reduction in harsh parenting practices in the total-PS score at post-intervention was detected in Mah et al. (2021) [34].

A reduction of over-reactivity at post-intervention (*p* = 0.020, ηp^2^ = 0.192), and at follow-up (*p* = 0.006, ηp^2^ = 0.264), and at verbosity at follow-up (*p* = 0.036, ηp^2^ = 0.165) and an overall significant total score at follow-up (*p* = 0.003, ηp^2^ = 0.302) was also reported [38]. Leitch et al. (2023) [30] and Van der Oord et al. (2012) [40] used the sub-scales over-reactivity and laxness/permissiveness, the former study [30] detected reductions in both dimensions at post-intervention (dZ = 1.11 and dZ = 1.02, respectively) and at follow-up (dZ = 1.16 and dZ = 0.77, respectively), whereas the latter study [40] did not report significant changes at post-intervention but found a significant reduction in over-reactivity at follow-up (*p* < 0.01, ES = 0.85).

The assessment of overreactive parenting showed significant improvements during the waitlist period and post-intervention (*p* ≤ 0.001), which was maintained at mid-term and 1-year follow-up (*p* ≤ 0.001 and *p* ≤ 0.01, respectively) in Bögels et al. (2021) [27]. One study [39] showed a significant reduction in overreactive parenting in mothers but a significant increase among fathers, and neither effect was significantly maintained at follow-up. One study [42] found no statistically significant differences in overactive parenting.

Another study assessed parenting with the Buri Parental Authority Questionnaire [24] and reported significant reductions in authoritarian and permissive parenting styles and an increase in authoritative parenting in the child-and-parent intervention group and in the parent-only group, with these effects maintained at follow-up.

Reliability analyses in Mah et al. (2021) [34] further indicated that parents in the intervention group were 4.73 times more likely to demonstrate clinically meaningful improvements in parenting practices than those receiving SBPT.

Several studies also considered parents’ behavior and ADHD symptoms. Some studies used the ADHD Rating Scale (ARS) [27,37,40]. A significant reduction in ADHD symptoms was reported at post-intervention (inattentiveness *p* < 0.01, ES = 0.36, and hyperactiveness/impulsiveness *p* < 0.05, ES = 0.48) and follow-up (*p* < 0.05, ES = 0.34 for inattentiveness and ES = 0.50 for hyperactiveness/impulsiveness) [40]. Similar results were detected in another study [27] at post-intervention and follow-up (*p* ≤ 0.001). Significant improvements in hyperactivity/impulsivity in the ITT approach (*p* < 0.001) were reported [37], but no improvement in inattentiveness at post-intervention nor at the follow-up. Per-protocol results showed significant improvement in inattentiveness (*p* < 0.05) and hyperactivity/impulsivity (*p* < 0.001), with maintenance of improvement in inattentiveness at long-term follow-up. This study also measured ADHD symptoms related to self-control showing significant improvements in ITT (*p* < 0.05) only at post-intervention. No significant difference in parental ADHD using the Adult ADHD Self-Report Scale (ASRS) was reported [30,33,41].

Regarding parents’ behavioral regulation, a significant improvement in the intervention group was reported (*p* = 0.027, η^2^ = 0.078) [34] whereas the control group showed significantly worse behavioral regulation after the intervention (*p* = 0.007 and η^2^ = 0.114). Furthermore, reliability analyses indicated that parents receiving MBPT had an 11.25 times higher likelihood of achieving clinically meaningful improvements in behavioral regulation than those in the standard training group. In the other study, parents’ attention, internalizing, and externalizing problems deteriorated during the waitlist period, and there was a subsequent significant improvement after intervention, maintained at follow-ups [27].

Positive changes in family functioning and sleep quality were reported, but similar changes were observed in the waitlist group, and no significant time-by-group interaction effects were observed [32]. Significant time effects on overall family functioning were reported [28], but post hoc comparisons did not reach significance, and there were no significant differences at follow-up. Additionally, a significant reduction in conflict at post-intervention (dZ = 0.74) and follow-up (dZ = 1.02) were detected but no significant change in the closeness domain of the Child–Parent Relationship Scale-Short Form (CPRS-SF) [30].

### 3.5. Risk of Bias

**Figure 2:**
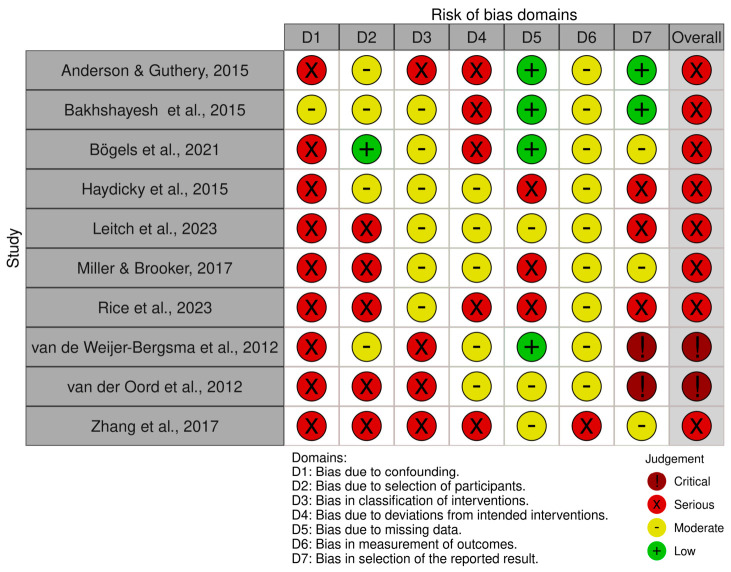
Risk of bias assessment of non-randomized studies [24,25,27,28,30,35,36,39,40,42] included in this systematic review, using the ROBINS-I tool.

Overall, the included non-randomized studies were predominantly rated as serious risk of bias (80%), with two studies rated as critical overall (Figure 2).

Among the assessed domains, bias due to missing data (D5) was mainly rated as a low or moderate risk (4/10 and 3/10 studies, respectively). Similarly, bias in classification of interventions (D3) and bias in measurement of outcomes (D6) were predominantly rated as moderate (6/10 and 9/10, respectively). High concern domains showed a predominantly serious risk, including bias due to confounding (D1) (9/10), bias due to selection of participants (D2) (5/10), bias due to deviations from intended interventions (D4) (5/10), and bias in selection of the reported results (D7) (2/10 critical, 3/10 serious) (Figure 2).

**Figure 3:**
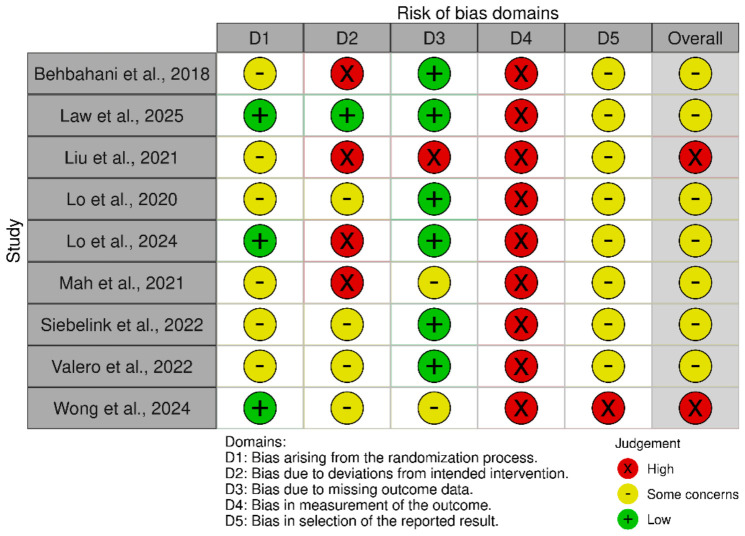
Risk of bias assessment of randomized trials [26,29,31,32,33,34,37,38,41] included in this systematic review, using the RoB2 tool.

Risk of bias assessments using the RoB2 tool indicated that seven studies (77.8%) were rated as some concerns and two studies (22.2%) as high overall risk of bias (Figure 3).

Across domains, bias arising from the randomization process (D1), deviations from intended interventions (D2), bias due to missing outcome data (D3), and bias in selection of the reported results (D5) were most often rated as low risk of bias or some concerns (D1—low 3/9; some concerns 6/9; D2—low 1/9; some concerns 4/9; D3—low 6/9; some concerns 2/9; and D5—some concerns 8/9), with isolated high risk of bias (Figure 3). In contrast, bias in measurement of the outcome (D4) was consistently rated as high risk of bias in all randomized trials (Figure 3).

### 3.6. Certainty of the Evidence (GRADE)

Overall, the quality of the evidence was rated as “moderate” or “very low”, depending on the study outcome (“stress” or “stress-related”) and design (RCT or non-RCT). Regarding specific domains of the assessment, risk of bias was rated as “serious” across all 9 RCT and 10 non-RCT studies evaluating both outcomes. Moreover, the inconsistency and indirectness domains were scored as “serious” in stress-related outcomes regardless of the study design, due to the heterogeneity of results and measurements (Table 5).

## 4. Discussion

The current systematic review suggests that MBI directed to parents of children with ADHD can be useful to decrease the well-known stress of the parental population [24,26,29,31,33,36,38]. The effects were sustained or even amplified at follow-up [27,28,30,37,40].

The magnitude of the effects of MBI varied across studies, probably due to the heterogeneity of the experimental design. For instance, some studies used MBI both for parents and children, which might have boosted stress reduction in parents by reducing child behavioral symptoms and subsequent parental challenges. Supporting this hypothesis mediation analysis [33] suggested that improvements in the child contributed to improvements in the parent outcomes. Another aspect to consider is the parent population included in the studies, namely the suggested differences in the effects on MBI in fathers and mothers. A significant stress reduction after MBI was detected only for fathers and not for mothers [39]. These findings might have been due to higher stress and specific ADHD in fathers than in mothers. Besides methodological constraints, clinical factors such as variable medication of children may account for the variable magnitude of the effects of MBI in managing stress in parents of ADHD children.

An important aspect across the results of this systematic review was that, among studies reporting significant post-intervention reductions in stress and including follow-up assessments, these beneficial effects were generally maintained over time, with statistical significance retained at follow-up in all such studies except Valero et al. (2022) [38]. Furthermore, some studies [27,28,30,37,40] showed delayed therapeutic benefits, with stress reduction becoming significant only at posterior follow-up assessments. Curiously, in one of the studies therapeutic effects only reached significance at one-year follow-up [27]. The mechanisms underlying these delayed effects deserve to be further investigated. It is possible that parents kept practicing mindfulness and integrating it into their routines, indicating that MBI may equip parents with tools to improve self-awareness and proper techniques for self-regulation. These results also indicate that it is important to manage parent expectations, as results may not appear during the intervention or at the end.

Some of the studies did not report statistically significant differences [32,34,35,41]. Variable durations of the MBI may have accounted for the results. Most studies with positive outcomes used the classical 8-week duration. Lo et al. (2024) [32] had the shortest intervention at 4 weeks and Mah et al. (2021) [34] had the longest MBI duration at 12 weeks, and these studies did not reach significance at stress scores. The classic 8 weeks format could be considered an optimal duration for the interventions. Another relevant factor is the type of MBIs which was mostly based in the classical MBSR program but also included different approaches, which may introduce some variations in the results. Other variables could have been introduced, namely the intervention performed during specific conditions, such as the COVID-19 pandemic [41].

A worsening in stress levels following MBI was reported in one non-RCT [42]. The authors propose that this may be due to the baseline assessment having taken place during summer vacation and the post-intervention assessment when the new school year had begun, leaving families more stressed with concerns about the academic life of their child and possibly interfering negatively with results. Therefore, the study design has some confounding factors that need to be considered.

Apart from stress, an analysis of secondary parameters—including quality of life, parenting behaviors, psychological well-being, and adult ADHD symptoms—reveals a nuanced picture of the impact of these interventions and seems to suggest that MBI may have a more comprehensive effect beyond direct stress level. In fact, the evidence regarding QOL leans toward overall improvements, or in specific domains rather than global shifts. Significant improvements in the social relations domain of the WHO-QOL-BREF were reported in some studies [29,36]. This may indicate that MBIs may enhance the relational aspects of a parent’s life. Improvements were also noted in psychological and environmental domains [29], though these were not universally replicated across all studies [36]. Since improvements were more pronounced in social domains [29,36], MBIs may be useful for parents struggling with social isolation or perceived lack of support.

The analyzed studies also indicate an improvement in well-being [33,37,41]. Changes in well-being were maintained at follow-up [41] whereas the other study [37] reported that while well-being improved post-intervention immediately, these gains were not maintained at long-term follow-up, suggesting a potential “washout” effect without ongoing practice.

Several studies reported psychological improvements, namely in psychological distress [30], and depressive symptoms [31] or anxiety [35]. Some studies reported improvements in mental health parameters in the intervention group, but also in the control group [31,32]. A study did not report significant changes in mental health [37]. The results in mental health are heterogeneous and need to be continued to ascertain if MBI may directly contribute to the mental health of parents and if this could be an indirect consequence of stress management.

One of the most robust findings across the reviewed studies is the reduction in dysfunctional parenting styles, particularly parental over-reactivity. Several studies [27,30,38] reported significant reductions in overreactive parenting, with effects often maintained or strengthened at the follow-up [40]. The consistent reduction in overreactive parenting [27,30,38] suggests that MBIs are particularly effective “de-escalation” tools. Clinicians may frame mindfulness not just as a relaxation technique, but as a functional intervention to reduce impulsive negative parental outbursts.

Reductions in parental “laxness” and conflict within the child–parent relationship [30] further support the role of mindfulness in stabilizing the domestic emotional climate. However, this needs to be studied in the future since studies fail to report significant improvements [38,40]. “Closeness” appears more resistant to change [30], implying that while MBIs reduce negative interactions, they may not immediately increase perceived intimacy. Furthermore, a reduction in harsh parenting [34] and in authoritarian and permissive parenting styles and an increase in authoritative parenting [24] were reported. It should be highlighted that results were not uniform in significance, with some studies reporting no significant difference for parenting style [42] and one study [39] showing reduced over-reactivity only in mothers and an opposite increase in fathers. These results may suggest that MBI help shift toward healthier parenting practices in the ADHD context, but this indication needs to be evaluated in the future.

Regarding the implications of MBI for parents with ADHD or high levels of symptoms, the results differ by study design and type of measurement, namely the scale used in the study. While some studies found significant reductions in parental ADHD symptoms (inattentiveness and hyperactivity) [27,37,40], other studies reported no significant differences compared to control groups [30,33,41]. MBIs should be viewed as a complementary rather than a primary treatment for parental ADHD, focused more on managing the emotional response to symptoms rather than the symptoms themselves.

Some other variables were explored to a lesser extent, such as sleep quality and family functioning, but their results were generally inconsistent or not statistically significant, which makes inferences about the real possible benefits of MBI in these outcomes uncertain.

### Limitations

This review also had some limitations. For instance, there was considerable heterogeneity in study design, interventions, report and measurement of outcomes across the included studies. Those are common limitations in cognitive-behavioral interventions using mindfulness [43]. Another limitation is a considerable risk of bias in the included studies. Most of the included non-randomized studies were predominantly rated as serious overall risk (80%), and most of the included randomized trials were rated as some concerns overall risk (77.8%), which undermines the strength of the conclusions that can be drawn. Consequently, the certainty of the evidence was scored as very low for stress-related outcomes and moderate for stress outcome, regardless of the study design.

It is also worth reflecting about the nature of mindfulness as an intervention, since MBI are self-referential by design and participants may present a change in their internal conceptualization of the outcome, rather than a change in the outcome itself.

This does not mean that the findings shown in this review are invalid, but it indicates that the interpretation should be conducted with caution and critical reasoning. Future research should include objective outcome measures such as physiological stress markers, behavioral observation of parent–child interaction, or informant-report measures, alongside the self-report instruments.

Other limitations that are common in research with MBI [43] are the need to include active controls. Studies using active controls (e.g., SBPT in Mah et al. 2021 [34]; CBT in Wong et al., 2024 [41]) often showed no significant difference between groups, whereas those using CAU controls (e.g., Law et al., 2025 [29]) showed robust effects. This suggests that some benefits attributed to mindfulness may be due to general “common factors” of therapy (such as social support and expert guidance) rather than mindfulness-specific mechanisms. Another limitation of the studies is the need to better determine if the effects of the MBI are permanent or transient.

It would also be advisable to use more objective tools in research. The reliance on self-report scales (like the ASRS or WHO-QOL) may introduce social desirability bias, where parents report improvements because they feel they should have improved. Furthermore, the use of diverse scales across studies (WHO-5 vs. WHO-QOL-BREF) makes direct comparison difficult. Finally, it should be noted that the studies varied in their focus, from general parental stress to parents with specific ADHD diagnoses. This variability likely accounts for the conflicting data regarding the impact of MBIs on core ADHD symptoms versus general psychological distress.

Even though there has been an increase in research on the topic of the efficacy of Mindfulness as a potential intervention for stress reduction in parents of ADHD children over the recent years, the research available is still sparse, with a lot of studies still in pilot form and small participation samples, and mostly concentrated in China and Western high-income countries, which comes as a possible barrier to generalization to the worldwide population with other cultural and healthcare backgrounds.

## 5. Conclusions

This systematic review indicates that MBI may be useful in the reduction in stress of parents of ADHD children, not only at the immediate moment of intervention but also at post-intervention. Additionally, it seems to possibly improve other factors that often surround stress, such as quality of life, parenting behaviors particularly over-reactivity, psychological well-being, and the parents’ own ADHD symptoms. These findings are consistent with theoretical models indicating greater emotional regulation and awareness with mindfulness practice [11,12], which can, in turn, help parents to adapt to the daily challenges they face. They must, however, be interpreted cautiously given the high risk of bias across the included studies and the preliminary nature of the evidence base.

The heterogeneity among results related to differences in intervention format, study design and power, children’s different medication uses and comorbidities across studies, measures of outcome, and follow-up duration contributed to substantial inconsistency across studies. This, combined with the high risk of bias identified, resulted in a downgrade in the evidence certainty in the GRADE analysis.

Further investigation is necessary to evaluate the current results which still remain not fully conclusive. New studies should prioritize using larger population samples, objective outcome measures and longer follow-up assessments to clarify results regarding the durability of effects or the eventual need for strategic “booster sessions”. Moreover, research in different worldwide healthcare settings is pertinent, as well as investigations directed at increasing father involvement and samples of parents of female children with ADHD, since most of the studied population has been mothers of ADHD sons.

## Figures and Tables

**Table 1:** Characteristics of the included studies organized according to the respective designs.

Study Design	Reference	Title	Country
Randomized Control Trial	Behbahani et al. (2018) [26]	Effects of Mindful Parenting Training on Clinical Symptoms in Children with Attention Deficit Hyperactivity Disorder and Parenting Stress: Randomized Controlled Trial	Iran
	Lo et al. (2020) [33]	The Effects of Family Based Mindfulness Intervention on ADHD Symptomology in Young Children and Their Parents: A Randomized Control Trial	China
	Liu et al. (2021) [31]	Applying the Mindful Parenting Program Among Chinese Parents of Children with ADHD: A Randomized Control Trial	China
	Mah et al. (2021) [34]	Mindfulness-Enhanced Behavioral Parent Training for Clinic-Referred Families of Children With ADHD: A Randomized Controlled Trial	Canada
	Siebelink et al. (2022) [37]	A randomized controlled trial (MindChamp) of a mindfulness-based intervention for children with ADHD and their parents	Netherlands
	Valero et al. (2022) [38]	Mindfulness Training for Children with ADHD and Their Parents: A Randomized Control Trial	Spain
	Lo et al. (2024) [32]	Effects of an online mindfulness-based program for parents of children with attention deficit/hyperactivity disorder: a pilot, mixed methods study	China
	Wong et al. (2024) [41]	The Effects of Mindfulness for Youth (MYmind) versus Group Cognitive Behavioral Therapy in Improving Attention and Reducing Behavioral Problems among Children with Attention-Deficit Hyperactivity Disorder and Their Parents: A Randomized Controlled Trial	China
	Law et al. (2025) [29]	The effectiveness of a mindfulness-based stress reduction (MBSR) program for parents of children with attention deficit hyperactivity disorder (ADHD): a pilot randomized controlled trial	China
Quasi- experimental study	Van der Oord et al. (2012) [40]	The Effectiveness of Mindfulness Training for Children with ADHD and Mindful Parenting for their Parents	Netherlands
	Bakhshayesh et al. (2015) [24] ^1^	The Effectiveness of Mindfulness Training for Children with ADHD and Parenting Styles of Parents	Iran
	Bögels et al. (2021) [27]	Family Mindfulness Training for Childhood ADHD: Short- and Long-Term Effects on Children, Fathers and Mothers	Netherlands
Pre–post intervention study	Van de Weijer-Bergsma et al. (2012) [39]	The Effectiveness of Mindfulness Training on Behavioral Problems and Attentional Functioning in Adolescents with ADHD	Netherlands
	Haydicky et al. (2015) [28]	Evaluation of MBCT for Adolescents with ADHD and Their Parents: Impact on Individual and Family Functioning	Canada
	Anderson and Guthery (2015) [25]	Mindfulness-Based Psychoeducation for Parents of Children with Attention-Deficit/Hyperactivity Disorder: An Applied Clinical Project	USA
	Miller and Brooker (2017) [35]	Mindfulness programming for parents and teachers of children with ADHD	Canada
	Zhang et al. (2017) [42]	Mindfulness-based interventions for Chinese Children with ADHD and Their Parents: a Pilot Mixed-Method Study	China
	Leitch et al. (2023) [30]	Co-designed Mindful Parenting for Parents of Children with ADHD: A Pilot and Feasibility Study	Australia
	Rice et al. (2023) [36]	Rolling out a mindfulness-based stress reduction intervention for parents of children with ADHD: a feasibility study	Ireland

^1^ Pre-posttest analysis intragroup, but different intervention groups were randomized and compared.

**Table 2:** Characteristics of the study populations included in the studies. Studies are ordered in accordance with Table 1.

Reference	Participants	
	Intervention Group	Control Group
Behbahani et al. (2018) [26]	*n* = 26 mothers (100% mothers)	*n* = 30 mothers (100% mothers)
	Higher education *n* = 8 (31%)	Higher education *n* = 9 (30%)
	
	Child (69% male)	Child (63% male)
	Age range 7–12	Age range 7–12
	Mean age 8.73 (SD ± 1.65)	Mean age 8.65 (SD ± 1.64)
	Medicated 100%	Medicated 100%
Lo et al. (2020) [33]	*n* = 50 parents (90% mothers)	*n* = 50 parents (86% mothers)
	Mean age 39.24 (SD ± 5.74)	Mean age 40.24 (SD ± 3.24)
	Married *n* = 45 (90%)	Married *n* = 46 (92%)
	Higher education *n* = 20 (40%)	Higher education *n* = 22 (44%)
	
	Child (70% male)	Child (88% male)
	Age range 5–7	Age range 5–7
	Mean age 6.24 (SD ± 0.87)	Mean age 5.92 (SD ± 0.70)
Liu et al. (2021) [31]	*n* = 58 parents (91% mothers)	*n* = 55 parents (89% mothers)
	Mean age 40.81 (SD ± 4.62)	Mean age 38.71 (SD ± 4.54)
	Married *n* = 55 (95%)	Married *n* = 51 (93%)
	Years of education 16.31 (SD ± 2.83)	Years of education 15.84 (SD ± 4.90)
	
	Child (78% male)	Child (76% male)
	Mean age 9.84 (SD ± 2.48)	Mean age 10.27 (SD ± 2.22)
	Medicated: Methylphenidate 19%, Atomoxetine 26%	Medicated: Methylphenidate 13%, Atomoxetine 11%
	ADHD-I 66%	ADHD-I 54%
	ADHD-H 3%	ADHD-H 6%
	ADHD-C 31%	ADHD-C 40%
Mah et al. (2021) [34]	*n* = 34 parents (94% mothers)	*n* = 29 parents (86% mothers)
	Mean age 41.0 (SD ± 7.4)	Mean age 40.9 (SD ± 7.3)
	Married/common-law 88%	Married/common-law 83%
	Higher education 88%	Higher education 90%
	
	Child (74% male)	Child (79% male)
	Age range 6–11	Age range 6–11
	Mean age 9.3 (SD ± 1.3)	Mean age 9.2 (SD ± 2.6)
	Medicated 44%	Medicated 34%
	ADHD-I 38%	ADHD-I 24%
	ADHD-H 6%	ADHD-H 3%
	ADHD-C 41%	ADHD-C 38%
	Subthreshold ADHD 15%	Subthreshold ADHD 35%
Siebelink et al. (2022) [37]	*n* = 55 parents (67% mothers)	*n* = 48 parents (69% mothers)
	Mean age 43.0 (SD ± 5.9)	Mean age 43.8 (SD ± 5.0)
	Married *n* = 41 (75%)	Married *n* = 39 (81%)
	Employed *n* = 47 (85%)	Employed *n* = 41 (85%)
	
	Child (71% male)	Child (69% male)
	Age range 8–16	Age range 8–16
	Mean age 11.0 (SD ± 1.8)	Mean age 11.4 (SD ± 1.8)
	Medicated 82%	Medicated 79%
Valero et al. (2022) [38]	*n* = 15 parents (of a total of 29 mothers and 1 father)	*n* = 15 parents (of a total of 29 mothers and 1 father)
	Mean age 44.6 (SD ± 5.1)	Mean age 47.4 (SD ± 3.81)
	Married/cohabitation *n* = 13 (87%)	Married/cohabitation *n* = 14 (93%)
	Higher education *n* = 8 (53%)	Higher education *n* = 8 (53%)
	Employed *n* = 11 (73%)	Employed *n* = 13 (87%)
	
	Child (73% male)	Child (80% male)
	Age range 9–14	Age range 9–14
	Mean age 10.33 (SD ± 1.83)	Mean age 11.6 (SD ± 1.29)
	Medicated 47%	Medicated 66%
	ADHD-I 27%	ADHD-I 33%
	ADHD-H 13%	ADHD-H 13%
	ADHD-C 60%	ADHD-C 53%
Lo et al. (2024) [32]	*n* = 23 parents (96% mothers)	*n* = 20 parents (90% mothers)
	Mean age 42.65 (SD ± 5.26)	Mean age 41.80 (SD ± 5.39)
	Married *n* = 20 (87%)	Married *n* = 16 (80%)
	Higher education *n* = 17 (74%)	Higher education *n* = 9 (45%)
	
	Child	Child
	Age range 6–18	Age range 6–18
	Mean age 10.80 (SD ± 3.33)	Mean age 9.53 (SD ± 2.98)
Wong et al. (2024) [41]	*n* = 69 parents (83% mothers)	*n* = 69 parents (83% mothers)
	Mean age 42.5 (SD ± 5.0)	Mean age 41.6 (SD ± 5.1)
	Married/cohabitation *n* = 56 (81%)	Married/cohabitation *n* = 57 (83%)
	Diploma or above *n* = 45 (65%)	Diploma or above *n* = 39 (57%)
	Employed *n* = 47 (68%)	Employed *n* = 42 (61%)
	
	Child (75% male)	Child (68% male)
	Age range 8–12	Age range 8–12
	Mean age 8.9 (SD ± 1.0)	Mean age 9.2 (SD ± 1.1)
	Medicated 51%	Medicated 42%
Law et al. (2025) [29]	*n* = 18 mothers (100% mothers)	*n* = 18 mothers (100% mothers)
	Mean age 37.5 (SD ± 5.63)	Mean age 34.17 (SD ± 4.79)
	Married/In stable relationship *n* = 11 (61%)	Married/In stable relationship *n* = 16 (89%)
	Higher education *n* = 2 (11%)	Higher education *n* = 8 (44%)
	Employed *n* = 2 (11%)	Employed *n* = 10 (56%)
	
	Child	Child
	Age range 3–12	Age range 3–12
	Medicated 39%	Medicated 50%
Van der Oord et al. (2012) [40]	*n* = 22 parents (96% mothers)	*n* = 11 parents
	High education *n* = 16 (73%)	

	Child (73% male)	
	Age range 8–12	
	Mean age 9.55 (SD ± 1.34)	
	Medicated 18%	
	ADHD-I 32%	
	ADHD-H 14%	
	ADHD-C 55%	
Bakhshayesh et al. (2015) [24]	*n* = 36 mothers (100% mothers)	

	Child (100% male)	
	Age range 6–12	
	Treatment 100% ^1^	
Bögels et al. (2021) [27]	*n* = 167 families (109 mothers (65%), 28 fathers, 30 both parents)	
	Child’s parents living together *n* = 96 (57%)	
	Higher education 27%, University 28%	

	Of which, 106 families (63%) participated in the waitlist assessment.	

	Child (62% male)	
	Age range 7–19	
	Mean age 11.4 (SD ± 2.27)	
	Medicated 43%	
	ADHD-I 34%	
	ADHD-C 52%	
	ADHD-NOS 5%, Other 3%, Missing 5%	
Van de Weijer-Bergsma et al. (2012) [39]	*n* = 19 parents (53% mothers)	

	Child (50% male)	
	Age range 11–15	
	Mean age 13.4	
	Medicated 10%	
	ADHD-I 40%	
	ADHD-H 10%	
	ADHD-C 50%	
Haydicky et al. (2015) [28]	*n* = 17 parents (94% mothers)	
	Married/cohabitating *n* = 13 (77%)	
	College program ~35%, Bachelor’s degree ~35%, Master’s degree ~18%	
	Employed ~83%	

	Adolescent (72% male)	
	Age range 13–18	
	Mean age 15.5 (SD ± 1.58)	
	Medicated 61%	
	ADHD-I 28%	
	ADHD-H 6%	
	ADHD-C 67%	
Anderson and Guthery (2015) [25]	*n* = 7 mothers (100% mothers)	
	Mean age 40.71	
	Married *n* = 5 (71%)	

	Child (43%)	
	Mean age 8.29	
Miller and Brooker (2017) [35]	*n* = 26 parents (69%) and teachers (31%), 85% female	
	Married/cohabitating 73%	
	Trade school/college 39%, University or some graduate training 19%, Graduate degree 27%	

	Child	
	Kindergarten grade 8	
Zhang et al. (2017) [42]	*n* = 11 parents (64% mothers)	
	Mean age 42.4 (SD ± 4.1)	
	Married *n* = 9 (82%)	
	Higher education *n* = 6 (55%)	
	Employed *n* = 8 (73%)	

	Child (72% male)	
	Age range 8–12	
	Mean age 9.5 (SD ± 1.4)	
	Medicated 91%	
Leitch et al. (2023) [30]	*n* = 18 parents (83% mothers)	
	Mean age 40.87 (SD ± 6.1)	
	Higher education *n* = 12 (67%)	
	Employed *n* = 14 (78%)	

	Child (83% male)	
	Age range 8–12	
	Mean age 10.29 (SD ± 1.20)	
	Medicated: Methylphenidate 50%, Clonidine 17%, Dexamphetamine 5%, Melatonin 22%, Fluoxetine 11%, Other 22%	
	ADHD-I 39%	
	ADHD-H 11%	
	ADHD-C 50%	
Rice et al. (2023) [36]	*n* = 29 parents	
	(23 mothers (73%) and 6 fathers, with both parents attending in 4 cases)	
	Mean age 48.88	
	Married *n* = 22 (88%)	
	Higher education *n* = 19 (76%)	

Percentages were calculated based on the *n*, when presented in the study. ^1^ Treatment was not specified.

**Table 4:** Summary table of main results. Studies are ordered in accordance with Table 1.

Reference	Stress		Stress-Related				
	Post-Intervention	Follow-Up	Well-Being/QOL	Mental Health	Self-Compassion	Parenting	Parental ADHD
RCTs (*n* = 9)							
Behbahani et al. (2018) [26]	+	+					
Lo et al. (2020) [33]	+		+				∅
Liu et al. (2021) [31]	+			+ Depression ± Anxiety	+		
Mah et al. (2021) [34]	∅					+	
Siebelink et al. (2022) [37]	∅	+	+	∅	+		±
Valero et al. (2022) [38]	+	∅				+ Over reactivity and verbosity ∅ Laxness	
Lo et al. (2024) [32]	∅	∅		∅			
Wong et al. (2024) [41]	∅	∅	+				∅
Law et al. (2025) [29]	+	+	±				
Non-RCTs (*n* = 10)							
Van der Oord et al. (2012) [40]	∅	+				+ Over reactivity ∅ Laxness	+
Bakhshayesh et al. (2015) [24]	+	+				+	
Bögels et al. (2021) [27]	∅	+				+ Over reactivity	+
Van de Weijer-Bergsma et al. (2012) [39]	± ^1^	± ^1^				± ^3^ Over reactivity	
Haydicky et al. (2015) [28]	± ^2^	+					
Anderson and Guthery (2015) [25]	+						
Miller and Brooker (2017) [35]	∅			∅ Depression + Anxiety			
Zhang et al. (2017) [42]	-					∅	
Leitch et al. (2023) [30]	∅	+		+		+ Over reactivity and laxness	∅
Rice et al. (2023) [36]	+		±				

+: Improvement; ±: Mixed results; ∅: Null/non-significant results; -: worsening. Stress-related outcomes improvements were marked as + when the result was significant at post-intervention, follow-up or both. ^1^ Significant stress reduction only for fathers and not for mothers. ^2^ No significant total stress score reduction but significant stress reduction in some areas. ^3^ Significant improvements among mothers but significant worsening among fathers.

**Table 5:** Certainty of the evidence assessment using GRADE.

Certainty of the Evidence								
Outcome	Studies (n)	Study Design	Risk of Bias	Inconsistency	Indirectness	Imprecision	Publication Bias	Certainty
**Stress**	9	RCT	Serious	Not Serious	Not Serious	Not Serious	Undetected	⊕⊕⊕
**Stress-related**	8	RCT	Serious	Serious	Serious	Not Serious	Undetected	⊕
**Stress**	10	Non-RCT	Serious	Not Serious	Not Serious	Not Serious	Undetected	⊕⊕⊕
**Stress-related**	10	Non-RCT	Serious	Serious	Serious	Not Serious	Undetected	⊕

RCT—randomized clinical trial; ⊕ Very low; ⊕⊕⊕ Moderate.

## Data Availability

Available upon reasonable request to the corresponding author.

## Abbreviations

The following abbreviations are used in this manuscript:

ADHD	Attention Deficit Hyperactivity Disorder
ADHD-I	Inattentive subtype of ADHD
ADHD-H	Hyperactive subtype of ADHD
ADHD-C	Combined subtype of ADHD
MBI	Mindfulness-Based Intervention
MBSR	Mindfulness-Based Stress Reduction
MBCT	Mindfulness-Based Cognitive Therapy
PRISMA	Preferred Reporting Items for Systematic Reviews and Meta-Analyses
RCT	Randomized Control Trial
ES	Effect size
ITT	Intention-to treat
CAU	Care As Usual
MBPT	Mindfulness enhanced Behavioral Parent Training
SBPT	Standard Behavioral Parent Training
CBT	Cognitive Behavioral Therapy
PSI	Parenting Stress Index
PSS	Perceived Stress Scale
ParSS	Parental Stress Scale
DASS-21	Depression Anxiety Stress Scale
SIPA	Stress Index for Parents of Adolescents
QOL	Quality of Life
WHO	World Health Organization
PS	Parenting Scale
ARS	ADHD Rating Scale
ASRS	Adult ADHD Self-Report Scale
CPRS-SF	Child–Parent Relationship Scale-Short Form

## Appendix A

**Table A1:** Search Strategy.

Pubmed Fields: Title, Abstract, MeSH Terms	Mindfulness AND (ADHD OR Attention Deficit Hyperactivity Disorder) AND (parent OR parents OR parental) AND Stress
Scopus Fields: Title, Abstract, Keyword	TITLE-ABS-KEY (Mindfulness) AND TITLE-ABS-KEY (ADHD OR Attention Deficit Hyperactivity Disorder) AND TITLE-ABS-KEY (parent OR parents OR parental) AND TITLE-ABS-KEY (Stress)
Web of Science Fields: Title, Abstract, Keyword	Mindfulness AND (ADHD OR Attention Deficit Hyperactivity Disorder) AND (parent OR parents OR parental) AND Stress
Cochrane Library Fields: Title, Abstract, Keyword	Mindfulness AND (ADHD OR Attention Deficit Hyperactivity Disorder) AND (parent OR parents OR parental) AND Stress

## Appendix B

**Table A2:** Data extraction variables.

Category	Data for Extraction
Study details	Study reference Year of publication Study type Location
ADHD children	Mean age Age range Gender Medication status ADHD subtype
Parents	Mean age Gender Marital status Employment Education
Mindfulness Based Intervention	Type of intervention Duration
Control	Type of control Duration
Outcome	Stress measure Stress-related outcomes measure Assessment timepoints Results
